# Endovascular Treatment of Internal Iliac Artery Aneurysms: Single Center Experience

**DOI:** 10.5935/1678-9741.20160023

**Published:** 2016

**Authors:** Rui Manuel Machado, Duarte Nuno Cunha Rego, Pedro Nuno Ferreira Pinto de Oliveira, Rui Manuel Gonçalves Fernandes de Almeida

**Affiliations:** 1 Hospital de Santo António - Centro Hospitalar do Porto, Porto, Portugal.; 2 Instituto de Ciências Biomédicas Abel Salazar (ICBAS), Porto, Portugal.

**Keywords:** Endovascular Procedures, Iliac Aneurysm, Embolization, Therapeutic

## Abstract

**Objective:**

Internal iliac artery aneurysms (IIAA) are rare, representing only 0.3% of aortoiliac aneurysms. Its treatment with open surgery is complex and associated with high morbidity and mortality, which led to increasing application of endovascular solutions. In this study, we aimed to evaluate outcomes of endovascular aneurysm repair (EVAR) of IIAA in one institution.

**Methods:**

We retrospectively reviewed all cases of IIAA treated with endovascular techniques between 2003 and 2014. Endpoints were morbidity, mortality, freedom from pelvic ischemic symptoms (buttock claudication, ischemic colitis, and spinal cord injury), and need for reintervention.

**Results:**

There were 16 patients, 13 males and 3 females, with mean age of 75.1±7 years. A total of 20 IIAA (4 cases were bilateral), with mean diameter of 37.9 mm, were treated. EVAR was performed in 13 (81.3%) patients, with associated internal iliac artery's outflow occlusion in 2. Iliac branch device was used in one patient. Two patients underwent endovascular IIAA embolization alone. One patient underwent percutaneous, transgluteal, IIAA embolization. IIAA flow preservation in at least one internal iliac artery was possible in 9 (56.3%) patients. Early mortality was 7% (1 case). Early morbidity was 18.8%. Pelvic ischemic complications occurred in 1 (7%) patient with buttock claudication. Late reintervention was needed in 3 patients, none of them for IIAA related complications.

**Conclusion:**

Endovascular treatment of IIAA is technically feasible and durable. Although overall morbidity is relatively high, major complications are infrequent and perioperative mortality is low. internal iliac artery flow preservation is technically challenging and, in a significant number of cases, not possible at all.

**Table:** 

Abbreviations, acronyms & symbols	
AAA	= Abdominal aortic aneurysm
EVAR	= Endovascular aneurysm repair
IBD	= Iliac branch device
IIA	= Internal iliac artery

## INTRODUCTION

Internal iliac artery aneurysms (IIAA) are a rare condition. Its prevalence is estimated as 0.03%^[1]^, representing 0.3% of all aortoiliac aneurysms and less than 20% of all isolated iliac artery aneurysms^[2]^. They are frequently asymptomatic and usually discovered during imaging examinations for aortic aneurysms or other conditions or at the time of rupture.

The elective treatment is justified by historical data, that reports a risk of rupture of up to 40% with a mortality rate of 80%^[3,4]^. Classical open repair of IIAA is technically challenging because of aneurysm localization and its outcomes confirm that complexity, with mortality rates of 11% for elective repair and 50% for emergency repair^[4]^.

Those results encouraged the development of endovascular solutions to treat this pathology and, as a result, in the last two decades, endovascular techniques have become the mainstay in IIAA's treatment^[5,6]^. However, this form of treatment comprises many different techniques without fully known outcomes in terms of rupture and ischemic complications prevention.

Endovascular treatment of IIAA can be based on a pure endovascular approach or a hybrid one as part of an endovascular aneurysm repair (EVAR) procedure to treat an aortoiliac aneurysm [compromising internal iliac artery (IIA) patency bilaterally], in which a bypass is performed, usually from the distal external iliac artery or the common femoral artery, to revascularize one of the internal iliac arteries^[7]^.

Pure endovascular techniques may be allocated in two main philosophies: with or without IIA flow preservation. While the second option is inarguably easier to perform, it can be associated with ischemic complications, especially in case of bilateral IIA occlusion. The worst of these complications include gluteal/perineal skin necrosis^[8,10]^, colon ischemia^[11,13]^, and spinal cord ischemia^[13,14]^. Despite representing a more benign complication, the incidence of buttock claudication can reach up to 28% in patients with unilateral occlusion of IIA and 42% in patients with bilateral IIA embolization^[15]^.

In order to achieve internal iliac arteries flow preservation, several endovascular techniques were developed, including external iliac to internal iliac artery stent grafts coupled with aorto-uni-iliac repairs, "sandwich" stent grafts, and iliac branch stent grafts (IBSGs)^[16]^.

Considering the low frequency of this pathology and the consequent scarcity of published data, we carried out this study to evaluate outcomes of EVAR of IIAAs performed in our institution and to assess the incidence of pelvic ischemic complications.

## METHODS

We retrospectively reviewed all cases of IIAA treated with endovascular techniques between 2003 and 2014. IIAA was defined as a focal dilation of the IIA (> 150% of its normal size), measured on imaging studies or documented at surgery. Demographics, clinical characteristics, and radiologic and operative data were obtained from the medical records.

Outcomes evaluated included mortality and cardiac, pulmonary, renal, and systemic complications. Acute renal failure in the postoperative period was defined as a change in serum creatinine of >0.3 mg/dL or the need for hemodialysis^[17]^. Pulmonary failure included patients requiring intubation for longer than 48 hours or respiratory failure requiring tracheostomy. Myocardial infarction was documented by elevation of biochemical markers. Pelvic ischemic complications were defined as acute colon ischemia, spinal cord infarction, skin necrosis and chronic buttock claudication. Endpoints recorded were 30-day and in-hospital mortality and major morbidity, need for reintervention and freedom from pelvic ischemic symptoms.

Statistical analyses were performed by the SSPS software (Statistical Package for the Social Sciences, version 20.0, SSPS Inc, Chicago, IL, USA). Continuous variables were presented as mean ± standard deviation and categorical variables were shown as frequency (percentage).

## RESULTS

From May 2003 to August 2014, 16 patients (13 males, 81.3%) underwent correction of 20 IIAA's. Demographic and clinical characteristics are described in Table 1. Of those patients, 13 (81.3%) had associated aortoiliac aneurysms, 1 (6.3%) patient had associated bilateral common iliac aneurysms without aortic aneurysm, and only 2 (12.5%) patients had isolated IIAAs. Four (25%) patients had bilateral IIAAs. Mean IIAA diameter was 37.9 mm (range 16-108 mm). Only one patient (with giant IIAAs) was symptomatic (constipation and hypogastric pain), all the others were incidental diagnosis.

**Table 1:** Patient's clinical data.

Variables	N (%)
Mean age (years)	75.1 ±7 (66-90)
Male	13 (81.3%)
Smoking	4 (26.7%)
Comorbidities	
Diabetes	2 (13.3%)
Hypertension	11 (73.3%)
Coronary heart disease	2 (13.3%)
COPD	1 (6.7%)
PAOD	39 (14.1%)
Aneurysm	
Isolated IIAA	2 (12.5%)
Common iliac aneurysm	1 (6.3%)
Aortoiliac aneurysm	13 (81.3%)
Treatment	
EVAR	13 (81.3%)
IIA coils embolization	1 (6.3%)
IIA plug occlusion	1 (6.3%)
IBD	1 (6.3%)
Coils embolization	2 (13.3%)
Percutaneous embolization	1 (6.3%)
Preservation of one IIA	9 (56.3%)

COPD=chronic obstructive pulmonary disease; PAOD=peripheral arterial occlusive disease; IIAA=internal iliac artery aneurysm; EVAR=endovascular aneurysm repair; IIA=internal iliac artery; IBD=iliac branch device

Institutional data reveals that IIAAs are present in 10.2% of the patients with aortic and/or iliac aneurysms^[18]^. We also observed that IIAAs were present in only 1.8% of the patients with aortic aneurysms whereas they were present in 45% of those with aorto-bi-iliac aneurysms (*P*<0.05). These data also showed that the association of IIAA and abdominal aortic aneurysm (AAA) at the time of the AAA's diagnosis was more frequent in patients aged over 70; however, this association was not statistically significant.

**Fig. 1:**
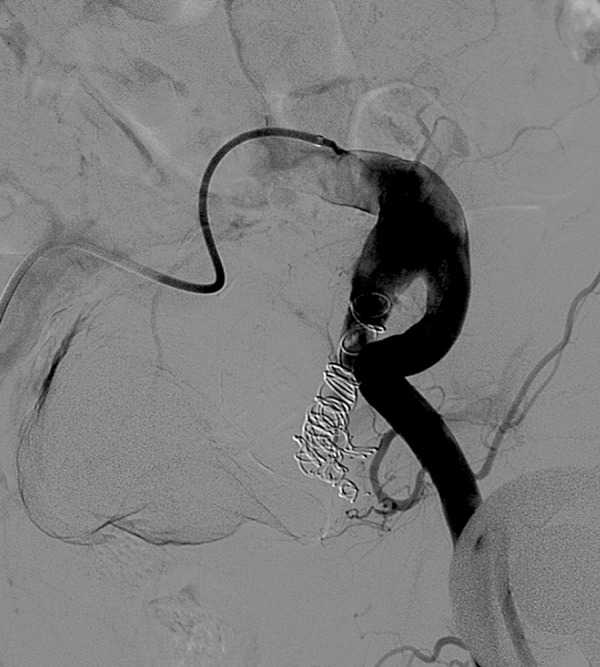
Internal iliac artery aneurysm embolization prior to EVAR.

**Fig. 2:**
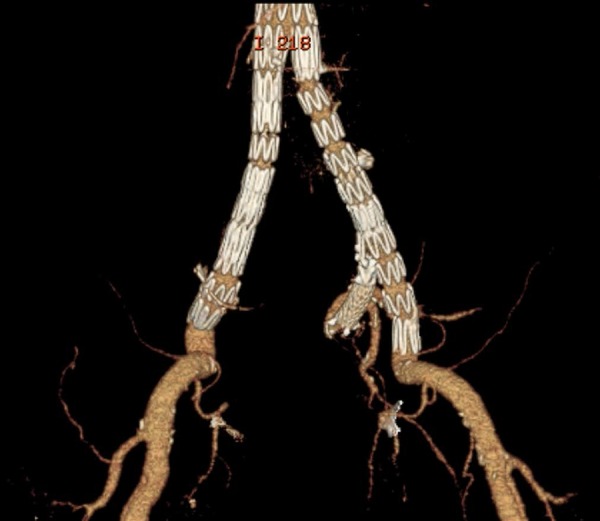
Left internal iliac artery aneurysm treatment and internal iliac artery revascularization with an iliac branch device.

**Fig. 3:**
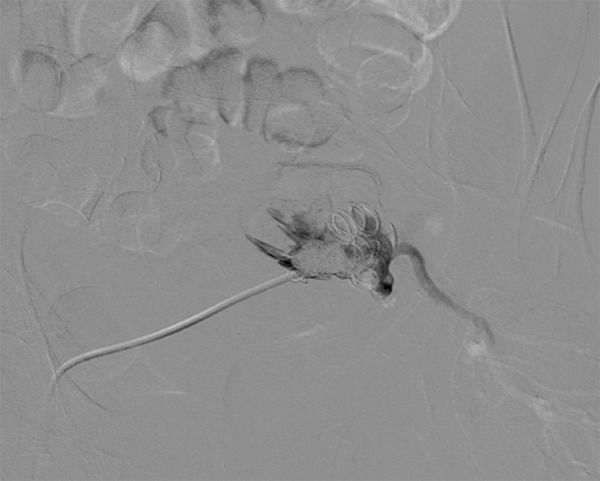
Echo-guided percutaneous, transgluteal, internal iliac artery aneurysm embolization.

The most frequent procedure was EVAR with limb extension to the EIA, which was performed in 13 (81.3%) patients. In 10 cases, only proximal coverage was performed while in 1 patient the procedure was associated with occlusion of one major branch of the IIA with a vascular plug. Another patient had his IIAA embolized with coils prior to EVAR (Figure 1) and another patient was treated with an iliac branch device (IBD); (Figura 2). Two patients underwent endovascular IIAA embolization with coils (bilaterally, in one case) without proximal coverage. One patient, who had undergone an abdominal aortic aneurysm (AAA) open repair 10 years earlier, with proximal ligation of the IAA due to a small aneurysm, presented with growth of his IIA and underwent percutaneous, ultrasound-guided, transgluteal, IIA embolization with coils (Figure 3).

In our sample, IIA flow preservation in at least one of the IIAs was possible in 9 (56.3%) patients, while 7 (43.8%) needed bilateral IIA occlusion (2 of those underwent staged repair).

There was one postoperative death, corresponding to a 30-day mortality of 6.3%. The patient died 3 days after endovascular embolization of a giant IIAA (108 mm), from unknown causes, while waiting for a proximal coverage procedure. Overall morbidity was 18.8%. Two patients developed acute renal failure (without the need for hemodialysis) and 1 patient, subjected to an aorto-bi-iliac EVAR, had a limb occlusion and was treated with a crossover femorofemoral bypass. Transfusional support was required in 5 patients (median of 2 units in the transfused patients). Median length of hospital stay was 6 days. Acute pelvic ischemic complications were not observed.

Mean follow-up after IIAA repair was 69±31 months. There were 3 late reinterventions: 2 patients presented with acute limb ischemia, 1 due to EVAR limb occlusion and another due to thrombosis of a crossover femorofemoral bypass in an aorto-uni-iliac EVAR. They were treated with a crossover femorofemoral bypass and thrombectomy of the bypass, respectively. The third late reintervention was in a patient with type Ib endoleak in a aorto-bi-iliac EVAR (contralateral to the treated IIAA) who underwent an iliac limb extension and consequent coverage of the other IIA (without pelvic ischemic complications). One case of buttock claudication was observed in a patient with bilateral IIA occlusion. Of the 16 patients treated, 9 underwent regular computed tomography follow-up in our institution. Mean imaging follow-up was 37±23 months. All but one patient showed stabilization/shrinkage of their IIAAs during follow-up. The patient had been treated with an EVAR (with extension to the external iliac artery) and occlusion of one major outflow artery of the IIA with a vascular plug; his IIAA aneurysm grew from 35 mm at baseline to 57mm on the 4^th^ year of follow-up. The patient died from cardiovascular cause before reintervention.

## DISCUSSION

IIAA's natural history is poorly known. Its average growth rate is about 4 mm/year^[19]^, similarly to that of AAAs. Several options of conventional surgery are possible: proximal ligation of the aneurysm (associated with late significant enlargement in nearly 50% of patients^[20]^, resection of the aneurysm with revascularization of its outflow, and proximal ligation with endoaneurysmorrhaphy. Endovascular treatment is a less invasive option that can, in many cases, be performed totally percutaneously. There are several options for endovascular treatment, the majority of them involving endoluminal occlusion of distal outflow branches (with coils or vascular plugs) in combination with aorta external iliac or common iliac to external iliac stent grafts. In some cases, proximal occlusion can be achieved with vascular plugs alone within the proximal neck. In patients with contralateral IIA occlusion or bilateral IIAAs where flow was sacrificed on one side, unilateral revascularization may be performed with iliac branch devices or open retroperitoneal bypass to the distal IIA with inflow from the distal external iliac or common femoral artery. Although classical series presented poor results with conventional therapy, a recent publication comparing efficacy and morbimortality of open and endovascular IIAA repairs showed no significant difference between them^[21]^.

Endovascular treatments have the disadvantage of not removing the mass effect induced by giant aneurysms.

IIAAs are usually diagnosed in association with aortoiliac aneurysms. In fact, isolated IIAAs frequently present as large, asymptomatic aneurysms or even with rupture. Our sample follows this epidemiologic pattern and our only casualty was an isolated, large, symptomatic aneurysm. Treatment is usually recommended for IIAAs over 3 cm, however, smaller aneurysms should be addressed when treating associated aortoiliac aneurysms; this was the case in 50% of our patients. Despite those traditional indications, a recent multicenter study revealed a low incidence of rupture in IIAAs smaller than 4 cm and the authors suggest that the threshold for elective treatment might be quite safely increased to 4 cm^[22]^.

Despite the occlusion of 2 IIAs in 43.8% of the cases, no acute, serious, pelvic ischemic complications were observed and only one patient had buttock claudication. These results contrast with the relatively high incidence of acute or chronic ischemic complications found by other groups^[15]^. However, this is a small series and our group stands by the policy of preserving IIA flow in all possible cases. Technological developments in the past years, with IBDs and better covered stents, have been a major help in achieving this goal, but IIA flow preservation remains a technical challenge difficult to overcome and, in our experience, a significant proportion of patients still needs occlusion of their IIA in order to achieve IIAA exclusion.

Our overall morbidity was relatively high; however, most of the complications were minor. Operative complications were infrequent, in contrast with classical series of open repair, which present frequent, serious, intraoperative complications with median transfusional requirements achieving up to 11 units per operation^[4]^. Although our transfusion rate was significant (31.3%), only one of the patients was transfused due to operative blood loss (the one with EVAR limb occlusion). The published results from other groups confirm low transfusional need with the endovascular treatment of IIAAs^[23]^.

Percutaneous Sono/computed tomography-guided embolization of IIAA allows intervention in patients that have previously been treated with proximal aneurysm ligation during open repair of AAA or proximal aneurysm coverage during EVAR. Several reports describe excellent technical success rates^[24,25]^. We describe one case in which these techniques were applied with good results.

## CONCLUSION

Our results show that endovascular treatment of IIAA is technically feasible and durable. In fact, during follow-up, in 20 treated IIAAs, only one patient needed reintervention for aneurysm growth. Although overall morbidity is relatively high, major complications are infrequent and perioperative mortality is low.

In our group's experience, IIA flow preservation is technically challenging and, in a significant number of cases, not possible at all. Nevertheless, based on larger series results, this technical endpoint should always be pursued to achieve the best clinical outcome.

**Table:** 

Authors' roles & responsibilities	
RMM	Conception and study design; execution of operations and/or trials; analysis and/or data interpretation; manuscript writing or critical review of its content; final manuscript approval
DNCR	Manuscript writing or critical review of its content; analysis and/or data interpretation; final manuscript approval
PNFPO	Conception and study design; analysis and/or data interpretation; statistical analysis; final manuscript approval
RMGFA	Conception and study design; execution of operations and/or trials; manuscript writing or critical review of its content; final manuscript approval
